# The Use of Probiotics in the Reduction of *Campylobacter* spp. Prevalence in Poultry

**DOI:** 10.3390/ani11051355

**Published:** 2021-05-10

**Authors:** Marcin Śmiałek, Joanna Kowalczyk, Andrzej Koncicki

**Affiliations:** Department of Poultry Diseases, Faculty of Veterinary Medicine, University of Warmia and Mazury, Oczapowskiego 13, 10-719 Olsztyn, Poland; welencasia@gmail.com (J.K.); koncicki@uwm.edu.pl (A.K.)

**Keywords:** poultry, *Campylobacter* spp., probiotics, prevention

## Abstract

**Simple Summary:**

According to European Food Safety Agency (EFSA), human campylobacteriosis is the most commonly diagnosed zoonotic disease in the EU. In 2018, the Member States reported 246,571 cases (30% increase since 2015). For years, poultry meat and poultry products have been considered a main source for human infections. In 2018, the highest occurrence of *Campylobacter* spp. was detected in chicken (37.5%) and turkey meat (28.2%). Considering this situation, there has been ongoing discussion regarding the potential strategies to minimize the level of *Campylobacter* spp. colonization in poultry and therefore in humans. In 2018, EFSA Panel on Biological Hazards indicated that use of feed and water additives is the second most likely strategy that can be successful in minimizing *Campylobacter* spp. colonization rate in broiler chickens. One of these water and feed additives are probiotics—living microorganisms which, when supplemented in the right dose, have a positive effect on microbial ecosystem of the host gut by ensuring a favorable balance between commensal and pathogenic microflora. In this review paper, the authors present current results of the studies concerning the potential use of probiotics as a preventive measure of *Campylobacter* spp. infection, under laboratory conditions and at a chicken farm level.

**Abstract:**

*Campylobacter* spp. are widely distributed microorganisms, many of which are commensals of gastrointestinal tract in multiple animal species, including poultry. Most commonly detected are *C. jejuni* and *C. coli*. Although infections are usually asymptomatic in poultry, poultry meat and products represent main sources of infection with these bacteria to humans. According to recent EFSA report, campylobacteriosis is the most commonly reported zoonotic disease. In 2018, EFSA Panel on Biological Hazards indicated that use of feed and water additives is the second most likely strategy that can be successful in minimizing *Campylobacter* spp. colonization rate in broiler chickens. One of those feed and water additives are probiotics. From numerous research papers it can be concluded that probiotics exhibit plenty of mechanisms of anti-*Campylobacter* activity, which were evaluated under in vitro conditions. These results, to some extent, can explain the efficacy of probiotics in in vivo studies, although different outcome can be observed under these two laboratory conditions. Probiotics are capable of reducing *Campylobacter* spp. population count in poultry gastrointestinal tract and they can reduce carcass contamination. Potential modes of anti-*Campylobacter* activity of probiotics, results of in vivo studies and studies performed at a farm level are widely discussed in the paper.

## 1. Introduction

Bacteria from the genus *Campylobacter* spp. are Gram-negative, widely distributed microorganisms, many of which are detected as commensals of the gastrointestinal tract (GIT) in multiple animal species, including poultry, domestic, and wild birds. *Campylobacter* spp., of which the two most common species are *C. jejuni* and *C. coli*, are thermophilic and microaerophilic bacteria that find favorable conditions for colonization in the GIT of all warm-blooded animals and birds, especially the latter due to their higher body temperature (approximately 41 °C) than other animal species [1].

Since the isolation of the genus *Campylobacter* from *Vibrio* spp. in 1963, *Campylobacter* spp. [2] infections have become the most important cause of foodborne bacterial gastroenteritis in humans in many developed countries [3]. For years, there have been an ongoing discussion and number of studies were performed in order to establish and evaluate potential strategies to overcome these issues. In this review paper, we have paid special attention to the potential of probiotics, which together with other food and water additives were jointly classified as the second most likely strategy capable of minimizing *Campylobacter* spp. prevalence in poultry by the European Food Safety Agency (EFSA) Panel on Biological Hazards [3].

## 2. *Campylobacter* spp. in Poultry—Colonization, Carcass Contamination, and Prevalence

*Campylobacter* spp. colonizations are more common in domestic than in free-living birds [4]. Although these bacteria do not pose a serious threat to birds per se, or to large-scale poultry production in general, colonizations with them are extremely important because poultry products represent the main source of *Campylobacter* bacteria in humans, and also because human campylobacteriosis has for many years been the most frequently diagnosed zoonotic enteropathy, surpassing even salmonellosis [3,5].

As thermotolerant microorganisms, *Campylobacter* spp., find favorable conditions for colonization in the gastrointestinal tract of birds, which have a higher body temperature than other animal species. The predominant species detected in birds include *C. jejuni* and *C. coli*, the latter having mainly been isolated from turkeys, but *C. lari* bacilli have also been sporadically isolated from chickens [6,7,8,9,10].

The colonization of the GIT of poultry with *Campylobacter* spp. usually proceeds without any distinct clinical manifestations and affects the small intestine, the cecum, and the cloaca. The consequences of colonization have been demonstrated to depend on the chicken breed and age. Humphrey et al. [11] have shown that the varying extents of the appearance of clinical symptoms in various chicken breeds can be ascribed to differences from breed to breed in the immune system responsiveness to infection with *Campylobacter* spp. They have also demonstrated that in some chicken breeds, the prolonged inflammatory reaction in the intestinal mucosa is due to a lack of interleukin (IL) 10 production, which, in turn, leads to diarrhea. Some works have also indicated that a clinical course of campylobacteriosis is most often noted in young birds [12,13] and manifests with enteritis and watery diarrhea, sometimes with mucus and blood in the excreta. This condition can lead to poorer body weight gains, reduced feed conversion ratio, and ultimately to differences in bird growth within the flock. Similar symptoms including diarrhea and lower body weight gains were observed in young turkeys [5]. In addition, cases of campylobacteriosis reported in flocks of laying hens weakened laying performance and egg hatchability [14].

The incubation period of campylobacteriosis in the cases with diarrhea was reported to range from 2 to 5 days [14]. The colonization of the intestines by these bacteria primarily takes place in the jejunum, then in the ileum, and finally in the cecum [15], where their population number peaks [16], and they can be detected and excreted in feces for a prolonged period [1]. *Campylobacter* spp. reaches higher concentration in distal parts of avian GIT [1]. For instance, the concentration of bacteria in the crop was significantly higher than in the gizzard [17], which results from growth pH requirements.

Even though *Campylobacter* spp. can be detected in the liver and other internal organs, deep muscles, and blood of infected birds [18], it is believed that the majority of incidences of contamination with these bacteria found in bird carcasses result from the contact of live birds or carcasses during slaughter with a contaminated external environment in a slaughterhouse or on the production farm. Hue et al. [6] demonstrated that the level of carcass contamination with *Campylobacter* spp. correlated directly with the degree of intestine invasion by these bacteria. In addition, Berrang et al. [19] demonstrated that the number of *Campylobacter* spp.-positive breast skin sponge samples increased after bird defeathering during slaughter from 1 (prior to defeathering) to 120 out of the 120 tested. Additionally, after defeathering, the *Campylobacter* spp. population count reached log_10_ 4.2 colony forming units (CFU) per sample. The same authors recorded an increase of *Campylobacter* spp.-positive breast skin samples from 0 to 13 out of 120 tested samples from an experimental group of slaughtered birds, the cloacae of half of which were plugged with tampons and sutured closed. According to these authors, an increase in the recovery of *Campylobacter* after defeathering can be related to the escape of contaminated feces from the cloaca during the process. Other risk factors increasing the likelihood of poultry carcass contamination include cross-contamination during transport, scalding, plucking, evisceration, and chilling operations [20,21,22]. Moreover, *Campylobacter* can survive on the surface of equipment used for bird slaughter despite cleaning and sanitizing, and the persistence of the bacteria can contribute to cross-contamination of carcasses during the slaughter process [23].

It has been demonstrated, that only 35 *Campylobacter* spp. CFU are sufficient to establish colonization in the bird gastrointestinal tract [24]. The transmission rate of *Campylobacter jejuni* was estimated to be 2.37 ± 0.295 infections per infectious bird per day. It means that in a flock consisting of 20,000 broilers, 95% of birds will become infected within 4.4–7.2 days after colonization of the first broiler [24]. The same study showed that the mean age at which birds become infected with *Campylobacter* spp. was 21 days of life. Based on selected papers published after the year 2000, it can be concluded that *Campylobacter* spp. prevalence in poultry flocks ranged from 3.5% to 71.5% [25,26]. Besides the immediate threat to consumers, such a widespread prevalence of *Campylobacter* bacteria in the poultry population poses an additional risk. Namely, that in a given location where large-scale production takes place, the constant presence of these microorganisms and the widespread use of chemotherapeutic agents facilitates the selection of *Campylobacter* spp. strains resistant to antimicrobials, which, unfortunately, translates to the results returned in monitoring studies [27,28]. For example, Woźniak and Wieliczko [29,30] showed an increase in the percentage of enrofloxacin-resistant *Campylobacter* strains isolated from poultry in Poland from 52.1% in 1994 to 93.6% in 2008. A similar trend was noted by these authors regarding resistance to tetracyclines. An additional disturbing aspect is the emergence at the beginning of the 21st century of multi-resistant *Campylobacter* strains that were not found in the 1990s [29,30]. These bacteria can pose a direct risk to consumers since these strains could be passed to humans via the food chain or by direct contact and they can additionally act as donors of antibiotic resistance genes to other bacteria.

In the light of the above and taking into account the EU policy to reduce the antibiotic usage in animal husbandry, it is worth emphasizing, that the use of antibiotics is not considered as a preventive option for *Campylobacter* spp. infections in poultry [3].

## 3. Transmission and Prevention of *Campylobacter* spp. in Poultry

Vertical route of infection with Campylobacter spp. in poultry is recognized not to be of great importance to Campylobacter spp. prevalence, despite these bacteria having been isolated from different parts of the reproductive system of laying hens [31,32,33]. This hypothesis is confirmed by studies which corroborated Campylobacter spp. genotype differences in the offspring of Campylobacter-positive breeder flocks [34,35].

Infection of birds with Campylobacter spp. occurs mainly horizontally through the gastrointestinal tract. The main sources and factors increasing the risk of infection in poultry include contaminated litter, rodents, flies, farm staff, other farm animals kept on or near the production farm, inadequately long production breaks, insufficient washing and disinfection of facilities, contamination of water and surrounding lands, proximity of Campylobacter-positive flocks, and thinning [3,36]. It is worth noting that in many cases, bacteria of the genus Campylobacter are present in livestock facilities even before birds are settled there. They were detected in dust and drinking water in poultry houses, which had been washed and disinfected immediately before the delivery of the chicks for rearing [36].

The findings from farm practice and location indicate the capacity of biosecurity measures to reduce the frequency and degree of Campylobacter spp. infection in poultry. However, taking into account the multitude of underlying factors of infection, it is reiterated increasingly often that these precautions should be comprehensive and multidimensional to raise the probability of achieving their goal. According to the recent report of the EFSA Panel on Biological Hazards regarding the control options for Campylobacter in broilers in primary production, the strategies most likely to be successful in minimizing the rate of infection and the prevalence of Campylobacter spp. in poultry products include vaccination, use of feed and water additives, discontinuation of thinning, employment of only a few and only well-trained staff, elimination of drinkers that allow standing water, addition of disinfectants to drinking water, hygienic anterooms, and designation of one set of tools per broiler house [3].

## 4. Campylobacteriosis Cases in Humans

An EFSA report states that the number of registered and confirmed European cases of human infection with *Campylobacter* bacteria in 2015 exceeded 190,000 [5]. Additionally, EFSA assumed that the approximate number of actual cases of infection could be as high as 9 million (because only 1 in 45 cases is confirmed in laboratory testing) [5]. The costs incurred in Europe associated with the decline in livestock productivity due to infection with *Campylobacter* bacteria together with the costs of treating infections with these bacteria in humans, have been estimated at approximately 2.4 billion EUR per year [5]. Additionally, the number of confirmed cases of infection with *Campylobacter* genus bacteria is increasing every year. According to the latest report by the EFSA and the European Centre for Disease Prevention and Control (ECDC), campylobacteriosis is the most commonly reported zoonotic disease in the EU. In 2018, the Member States reported 246,571 cases (an approximate 30% increase over the number in the 2015 report) [3].

The widespread prevalence of *Campylobacter* spp. in the animal population carries the risk of contamination of food products such as raw meat, milk, and water. The available literature data show that poultry and poultry meat are considered to be a common source of *Campylobacter* spp. infections; however, beef and pork products are also emphasized to contribute to the unfavorable epidemiological situation of campylobacteriosis in the human population. Considering the 2015 data on campylobacteriosis [5], broiler chickens and products made of them accounted for nearly 22.5%, eggs and egg products for 6.12%, but milk or cattle and beef products for less than 4.1% of cases each [5]. In the 2018 EFSA report, the highest occurrence of *Campylobacter* spp. was detected in chicken meat (37.5%) and turkey meat (28.2%) [3].

## 5. Benefits from Using Probiotics in Poultry

Probiotics are living microorganisms which, when supplemented in the right dose, have a positive effect on the microbial ecosystem of the host gut by ensuring a favorable balance between commensal and pathogenic microflora [37].

The bacteria most often used as probiotics include those from the following genera: *Bacillus, Bifidobacterium, Enterococcus, Lactobacillus, Pediococcus*, and *Streptococcus*. Probiotics are also produced from selected species of fungi and yeasts (e.g., *Saccharomyces*).

The main beneficial effects of probiotics relate primarily to their raising of feed digestibility and bioavailability, stimulation of the immune system, improvement of health, and provision of superior organoleptic properties and chemical composition of carcasses [38,39,40,41,42,43,44,45,46].

One of the first reports on the beneficial effects of probiotics in poultry comes from 1973, when Tortuero [47] noticed an improvement in weight gain coinciding with the use of *L. acidophilus* in chicks during the first 5 days of life. In addition, in this experiment, the group receiving the probiotic was characterized by *Lactobacillus* dominance among the gastrointestinal microflora and by the simultaneous reduction in enterococci population. Considering the enterococci population in the probiotic-treated group, Tortuero [47] obtained a result similar to that in the group of birds receiving the antibiotic instead of the *Lactobacillus* culture in the same period. In addition, Nurmi and Rantal [48] showed that protection against *Salmonella infantis* could be obtained in broiler chicks by the per os administration of bacterial flora isolated from the intestines of healthy adult chickens. This concept was later referred to as competitive exclusion.

Probiotic bacteria are able to inhibit the growth of pathogenic microflora in the gastrointestinal tract of birds. This is due to the depletion of nutrients in the environment, the blocking of target receptors for pathogens on the surface of epithelial cells, or the production of natural antibacterial agents known as bacteriocins [49]. Probiotic bacteria also exhibit strong immunomodulatory effects, improving the local immune mechanisms in the gastrointestinal tract. For example, their regular and occasional uses in poultry have been shown to have an immunostimulating effect on interferon production; activities of macrophages, heterophiles, lymphocytes, and natural killer (NK) cells; and the production of specific antibodies [38,39,41,42,44]. In addition, it was previously concluded that probiotics exert a non-specific effect on the stimulation of the gut-associated lymphoid tissue (GALT), but as antigens with relatively low immunogenicity, they do not contribute to the excessive development of the inflammatory reaction nor activate the immunological mechanisms aiming at their complete elimination [43]. Through these properties, probiotics enhance the responsiveness of the immune system to an infecting pathogen [43]. These phenomena induced by probiotics minimize the risk of colonization or limit the population size of a wide range of microorganisms potentially pathogenic to poultry [7,10,13,41,48,50,51,52,53,54].

## 6. Probiotics and Poultry *Campylobacter* spp. Infection and Colonization

Various systems are used to assess probiotic efficacy in minimizing the consequences of infections with *Campylobacter* spp., and they have been previously reviewed [10,37,55]. In this review article, we would like to present the general scope of probiotics’ modes of action against *Campylobacter* from molecular, in vitro, and in vivo studies and in conclude the work to present the results obtained in field experiments performed under commercial broiler farm conditions. General modes of probiotic anti-*Campylobacter* activity are summarized in Table 1.

After entering the avian GIT, *Campylobacter* spp. use different mechanisms to establish their population in the gut environment, like motility, chemotaxis, adhesion, intracellular infection, and the capability to synthesize entero- and cytotoxins, as reviewed by Mohan [10]. Given the set of mechanisms, the bacteria can exploit, most research works addressing the choice of potential probiotic bacteria have been focused on the evaluation of their potential to overcome these properties of *Campylobacter* spp.

## 7. In Vitro Studies

Most of the experimental models applicable to the in vitro assessment of probiotic efficacy refer to their ability to inhibit the proliferation potential of *Campylobacter* spp. in co-cultured assays or their colonization potential in cell cultures.

Contemporary scientific research has in the most past assumed the use of lactic acid bacteria (LAB; particularly various selected strains of *Lactobacillus* genus) as probiotics against *Campylobacter jejuni* infection. From a number of pertinent studies, it can be concluded that LAB alone, or in the mixture with different carbohydrate prebiotics, can decrease the *C. jejuni* growth rate by 4–8 log_10_ CFU/mL after 24–72 h of co-culturing [65,66]. The main mechanism of the inhibitory effect of LAB against *Campylobacter* growth has been described in those studies as acidification of the medium by producing lactic and acetic acids [56,57,58,62]. In an assortment of studies, the strongest antagonism towards *Campylobacter* was exhibited by *L. salivarius* and *L. reuteri*. It has also been confirmed in a study by Dec et al. [56], who evaluated the probiotic potential of 46 *Lactobacillus* isolates from chicken feces or cloacae against *C. jejuni* and *C. coli*. Singled out again in this research, *L. salivarius* and *L. reuteri* evinced the highest anti-*Campylobacter* activity, and results indicated that it was the reduced pH of the supernatant from *Lactobacilli* culture that played the key role in inhibiting *Campylobacter* growth. The cited authors also highlighted that all the isolated *Lactobacillus* strains were capable of producing hydrogen peroxidase, but the production rate did not correlate with the level of anti-*Campylobacter* properties [56], which has been also demonstrated previously by Campana et al. [67], and it is a result of catalase production by these *Campylobacter* species [67].

Both acidic and neutralized lactobacilli supernatants have been shown to inhibit *C. jejuni* growth to comparable levels. This finding suggests that other bioactive factors could also contribute to pathogenic bacteria growth inhibition. Messaoudi et al. [60] described *L. salivarius* isolates that were capable of producing bacteriocins and exhibited high anti-*Campylobacter* activity. Bacteriocins are small proteins produced by bacteria including LAB, which enable them to inhibit the growth of other bacteria in the environment. The inhibitory effect of bacteriocins against *Campylobacter* spp. growth was confirmed in both in vitro and in vivo studies [61]. Bearing this in mind, it seems that bacteriocin production and acidification can play equally important roles in inhibiting the growth of pathogenic bacteria.

It has been demonstrated that the mixture of bacterial stains of the *Lactobacillus* genus that have been individually confirmed to inhibit the growth of *Campylobacter* in in vitro studies may not display additive properties under the same conditions, which has been suggested in the in vivo studies. A recent study by Taha-Abdelazi et al. [62] evaluated the ability of five different *Lactobacillus* strains (*L. salivarius*, *L. johnsonii*, *L. reuteri*, *L. crispatus*, and *L. gasseri*) to inhibit *Campylobacter jejuni* growth. In this study, the authors confirmed the efficacy of all strains and reported the highest inhibitory activity for *L. salivarius*. However, its activity was comparable to the activity of a mixture of all five strains tested. This suggests some limitations in the possibility of direct translation of in vitro study results into in vivo study findings or may indicate that probiotics used in a mixture can be antagonistic to each other.

Another step toward identifying the mechanisms of probiotic action against *Campylobacter* is made via investigations exploring changes in the molecular properties of pathogenic bacteria and their capability of adhering to or invading the target cells. However, the applicability of these investigations to poultry campylobacteriosis is less than perfect because of their use of human and not avian cell lines, as the latter are commercially unavailable [55]. This limitation coupled with the only selectively aspectual observation of the pathogen–host interaction that is possible can hinder extrapolation of their results into likely results under farm conditions.

However, it has been demonstrated that *L. salivarius*, *L. johnsonii*, *L. crispatus*, and *L. gasseri* significantly reduced the expression of virulence-related genes in *C. jejuni* after 24 h of co-incubation with probiotic bacteria. This down-regulation involved *C. jejuni* genes responsible for both motility (*flaA*, *flaB*, and *flhA*) and invasion (*ciaB*), which correlated with less extensive invasion by *C. jejuni* of Caco-2 human intestinal epithelial cell line by over 80% [62]. Using the same cell line, it has been demonstrated that *L. acidophilus* was capable of reducing both adhesion of 10 various human-origin *C. jejuni* strains to the intestinal epithelial cells and these cells’ invasion by the bacteria [61]. In this experiment, the authors used different schemes of probiotic treatment of Caco-2 cells, which were referred to as exclusion (probiotic treatment prior to infection), competition (probiotic treatment and infection performed simultaneously), and displacement (probiotic treatment after the infection). In all three strategies, the authors reported diminished adhesion and invasion of most of the *C. jejuni* strains used, but the most prominent results were achieved in the displacement test where the adhesion was reduced by 11–53% and the invasion by 11–52% [67]. These results could be explained by competitive exclusion where the LAB block the adhesion sites on epithelial cells for *C. jejuni* and/or by probable bacteriocin production by the *L. acidophilus* probiotic.

The adhesion of probiotic bacteria to epithelial cells as one of their mechanisms of anti-*Campylobacter* activity has been suggested previously [10]. Properties of probiotic bacteria that impart their adherent capacity include their high hydrophobicity as well as specific binding to cells by surface adhesins. Wang et al. [68] demonstrated that the high hydrophobicity of *L. casei* strain ZL-4 correlated with high inhibiting activity against the adhesion of *C. jejuni* to intestinal cells of culture HT-29 and the invasion of these cells by the bacteria. In another study, Dec et al. [56] demonstrated that approximately 98% (45 out of 46 tested) of different *Lactobacilli* strains of chicken origin that possessed activity against *C. jejuni* and *C. coli* of chicken origin displayed very high hydrophobicity.

Recently, in parallel to works investigating the effect of probiotics on the growth and properties of *Campylobacter* spp., probiotic bacteria have also been tested for their immunomodulatory and immunostimulatory properties. Despite the studies conducted so far on the specificity of poultry immune system response to infection with *Campylobacter* spp. being few, their results enable a much better perception of the potential efficacy of the tested probiotics under field conditions. In a recent study performed by Taha-Abdelazi et al. [62], the stimulation of macrophages with either a single species or a species mixture of heat-killed lactobacilli (*L. salivarius*, *L. johnsonii*, *L. reuteri*, *L. crispatus*, and *L. gasseri*) enhanced *C. jejuni* phagocytosis. At the same time, macrophages exposed to lactobacilli had increased expression of interferon-γ, IL-1β, IL-12p40, and IL-10 genes. Furthermore, *L. salivarius*, *L. reuteri*, *L. crispatus*, and lactobacilli mixture increased the expression of the co-stimulatory CD40, CD80, and CD86 molecules in macrophages [62]. Additionally, it has been shown that B lymphocytes are involved in *C. jejuni* clearance and decrease the shedding of the bacteria [15]. Co-stimulatory molecules are known to participate in the cascade of antigenic signal transduction and activation of both T and B cells. Therefore, it may be speculated that the LAB mixture can enhance both the non-specific and specific immune responses against *Campylobacter*.

## 8. In Vivo Studies

At this stage, it can be summarized that probiotic bacteria possess and display, under in vitro conditions, multiple mechanisms of anti-*Campylobacter* activity. In vivo tests allow for a more comprehensive evaluation of probiotics and their efficacy in the highly variable and complex environment of the chicken GIT. These tests can be divided into those in which the efficacy of probiotics is assessed against natural *Campylobacter* spp. infection and those assaying how they act against artificial infection.

The choice of probiotic bacteria for experimental and commercial needs is governed by strict criteria of safety, functionality, and technological feasibility. One of the basic commercial criteria is that they should have confirmed efficacy in the target animal species [68]. Although most often the primary selection and determination of the basic properties of probiotic strains are based on in vitro studies, most of the criteria described above can only be assessed using in vivo tests. It is worth mentioning here that not always can an effect in one research strategy be directly replicated in another. Robyn et al. [69,70] demonstrated that the *Enterococcus faecalis* strain MB 5259 that was capable of inhibiting *C. jejuni* growth in in vitro tests was unable to do so in in vivo experiments, irrespective of the inoculum volume. On the other hand, in these studies *E. faecalis* was capable of colonizing the chicken GIT despite its impairment during passage through the tract which the in vitro analysis showed [69,70].

Fritts et al. [63] demonstrated that the Calsporin^®^ probiotic comprising *Bacillus subtilis* (strain C-3102) administered to birds in feed from the day of hatching to slaughter (at 42 days) was capable of reducing the extent of broiler carcass *Campylobacter* contamination in the course of natural infection. During the experiment, the probiotic-treated and control birds were kept in isolated pens and separated from each other in a way that prevented cross-contamination with probiotic bacteria. In two experiments, the authors recorded a 6.5% reduction in mean *Campylobacter* spp. CFU/mL after probiotic administration. In the same study, the population counts of *Salmonella* and *E. coli* decreased in carcass samples of the probiotic-treated group of chickens. The results of those studies were also confirmed for the ceca of broilers, as Guyard-Nicodème et al. [71] demonstrated a decrease in *C. jejuni* count in birds given Calsporin^®^ after artificial *C. jejuni* infection. On the other hand, Garcia-Hernández et al. [72] did not record any reduction of the *C. jejuni* population count in the ceca of chickens artificially infected at 14 days of life after a similar treatment with probiotic *B. subtilis* of the DSM17299 strain.

Saint-Cyr et al. [49] demonstrated that *L. salivarius* of the SMXD51 strain given to birds on the first day of life and afterwards every 2–3 days was capable of reducing the *C. jejuni* count after artificial infection at 11 days of life. The bacterial populations in the cecal content on the 14th and 35th days of the birds’ life declined by 0.82 and 2.81 log_10_, respectively. This was also confirmed by Ghareeb et al. [13], who treated the birds with a multispecies probiotic comprising *Enterococcus faecium*, *Pediococcus acidilactici*, *Bifidobacterium animalis*, *L. salivarius*, and *L. reuteri* at doses of 2 or 20 mg/bird via drinking water from the first day of life and noted up to 5.81 and 5.85 log_10_ reduction of *C. jejuni* CFU/g in the cecal content after the birds were artificially infected on the day of hatching. These data may suggest that a combined preparation may display higher anti-*Campylobacter* activity than single strain probiotics.

The works by Saint-Cyr et al. [49] and Ghareeb et al. [13] cited above are only selected examples confirming the anti-*Campylobacter* activity of probiotics, while this efficacy has also been confirmed in many other scientific studies [13,54,73,74]. In contrast, studies have also reported the inefficacy of probiotics in this context [69,75,76]. Nevertheless, as in the case of in vitro studies, distinctly different results may also be obtained in in vivo studies evaluating the benefits of probiotics against poultry campylobacteriosis under controlled experimental conditions. On the other hand, it is known that the results of in vitro tests will not always translate into comparable results under in vivo conditions [69,70]. This may be due to differences in the bacterial strain used as a probiotic, the final composition of the probiotic, its dose and application pattern, the *Campylobacter* strain used in the experiment, experimental conditions, the age and breed of the birds, and therefore different outcomes of probiotic activity. Additionally, it is worth emphasizing that in vitro studies do not take into account the complexity and variability of birds GIT environment as well as the interactions between probiotic, *Campylobacter* spp., and GALT that may occur in vivo.

## 9. On Farm Studies

In the case of experiments carried out under commercial poultry farms conditions, an additional set of variables that were not included in laboratory experiments should be taken into account and they include, i.a.: differences in farm infrastructure; breeding practices; epidemiological status of the farm/region; biosecurity regime; season of the year; quality, health, and immunological status of chicks; preventive vaccination program; antibiotic treatment administered to birds; and the use of other water and feed additives, which may directly affect either the probiotic or the *Campylobacter* spp. population and others. Therefore, it is not surprising that currently there are scarce research works describing field experiments on the anti-*Campylobacter* activity of probiotics in poultry. Although, Śmiałek et al. [64] demonstrated that a multispecies probiotic comprising an LAB mixture (*L. lactis*, *Carnobacterium divergens*, *L. casei*, and *L. plantarum*) and *Saccharomyces cerevisiae* given to birds in feed for the entire production cycle was capable of reducing the *Campylobacter* spp. population count in broiler ceca and feces. In this case, the *Campylobacter* spp. count was approximately 10 times lower in experimental chickens than in the control chickens. The authors also recorded no growth of *Campylobacter* spp. from samples of pectoral muscles and overlying skin after slaughter of the probiotic-treated birds, while 50% of the control bird sample cultures were positive, with a mean *Campylobacter* spp. count of 4.15 × 10^1^ CFU/g. Those researchers compared the results of this experiment to the results of control production cycles in which the population number of *Campylobacter* spp. from equivalent samples from the same farm and the same chicken houses was monitored. The production cycle prior to the experiment and two production cycles after the experiment were used as these controls. In all of them, the *Campylobacter* spp. population count was significantly higher in the chicken house in which the probiotic was used during the main experiment. The pre-experiment production cycle results allowed the most appropriate chicken house to be selected in which the probiotic was used and the full set of determined dependencies enabled the authors to conclude that the probiotic used as a feed additive in the study was capable of reducing the extent of *Campylobacter* spp. invasion in the GIT and the contamination level in the birds’ environment, which subsequently contributed to the improved hygienic parameters of the analyzed poultry carcasses [64].

## 10. Final Conclusions

The use of probiotics is one of the strategies that can be implemented in order to minimize the risk of infection and the level of colonization with *Campylobacter* spp. in GIT of chickens and humans. It has been demonstrated that numerous potential probiotic strains, or probiotic strains mixture, are capable of inhibiting *Campylobacter* spp. growth in in vitro studies. In the nearest future, a special attention should be paid to in vivo studies. The results of those studies would enable the evaluation of in vitro studies results, evaluation of probiotics modes of action as well as the verification of target probiotic composition, which in turn would result in optimization of probiotic formula in order to establish commercial products with the highest anti-*Campylobacter* activity in poultry.

Additionally, it is worth to emphasize the lack of knowledge into the immunomodulatory properties of probiotics, in the context of both: general immunity enhancement as well as the specific anti-*Campylobacter* immunity stimulation.

Considering the list-topping position of poultry and poultry products for many years as potential sources of *Campylobacter* spp. to humans, it should be expected that in the near future, adequate administrative programs to alleviate the peril in this situation will be implemented. Considering the above it is necessary to conduct appropriate studies under farm conditions in order to evaluate the efficacy of probiotics in minimizing the risk of *Campylobacter* spp. infection and infection rate. In these studies, probiotics should be considered as one of the variable elements of multidimensional biosecurity program.

## Figures and Tables

**Table 1:** Potential mechanisms of anti-*Campylobacter* activity of probiotics, established based on selected in vitro and in vivo studies.

Probiotic Strain(s) (Origin)	Mode of Action (Experiment Conditions)	Result (Summarized Based on Different References)	References
Different *Lactobacilli* (chicken) Different LAB strains (9 strains from environmental samples of chicken farms; others—not specified) Different *Lactobacilli* strains (human)	Organic acids production (in vitro)	Reduced pH inhibits *Campylobacter* growth	Dec et al. [56] Dubois Dauphin et al. [57] Fernández et al. [58]
Different *Lactobacilli* (chicken)	Hydrogen peroxidase production (in vitro)	Suggested to be involved in antimicrobial activity of probiotics	Dec et al. [56]
Different *Lactobacilli* (chicken) Different LAB strains (fermented pickles, health infant feces and fermented dairy products)	Hydrophobicity (in vitro)	Suggested to correlate with probiotic adhesion to intestinal cells ability and therefore competitive exclusion	Dec et al. [56] Wang et al. [59]
Different LAB strains (chicken)	Bacteriocins production (in vitro)	Direct anti-*Campylobacter* activity	Messaoudi et al. [60]
*Lactobacillus acidophilus* ATCC 4356 (human) Different *Lactobacilli* (chicken)	Attenuation of *Campylobacter* (in vitro; cell lines) Adhesion of probiotic strain to epithelial cells (in vitro; cell lines)	Decreased expression of *Campylobacter* virulence related genes	Campana et al. [61] * Taha-Abdelaziz et al. [62]
		Decreased *Campylobacter* adhesion to human intestinal epithelial cells by over 30%	
		Decreased *Campylobacter* invasion into human intestinal epithelial cells by over 80%	
*Lactobacillus acidophilus* ATCC 4356 (human)	Therapeutic properties (in vitro; cell lines)	Displacement of *Campylobacter* by probiotics in human intestinal epithelial cells	Campana et al. [61]
*Bacillus subtilis* C-3102 (Calsporin^®^) *Lactobacillus salivarius* SMXD51 (chicken) Different LAB strains and *Saccharomyces cerevisiae* (chicken, turkey, carp, and plant silage)	Modulation of gut environment (in vivo; on farm studies; broiler chickens)	Decreased population of *Campylobacter* in GIT and/or on the carcass in vivo	Fritts et al. [63] Saint-Cyr et al. [49] Śmiałek et al. [64]
Different *Lactobacilli* (chicken)	Immune system stimulation (in vitro; cell lines)	Enhanced macrophages phagocytosis ability of *C. jejuni*. Immunomodulation.	Taha-Abdelaziz et al. [62]

* Gene expression was not evaluated in the paper by Campana et al. [59].

## Data Availability

The datasets used and/or analyzed during paper preparation are available from the corresponding author on reasonable request.
